# The prevalence of hyperuricemia in China: a meta-analysis

**DOI:** 10.1186/1471-2458-11-832

**Published:** 2011-10-27

**Authors:** Liu B, Wang T, Zhao HN, Yue WW, Yu HP, Liu CX, Yin J, Jia RY, Nie HW

**Affiliations:** 1 Jinan Institute of Cardiovascular Disease, The Fourth People's Hospital of Jinan, Shifan Road, Jinan, 250031, China; 2 Department of Public Health, The Second Affiliated Hospital of Soochow University, Sanxiang Road, Suzhou, 215000, China

## Abstract

**Background:**

The prevalence of hyperuricemia varied in different populations and it appeared to be increasing in the past decades. Recent studies suggest that hyperuricemia is an independent risk factor for cardiovascular disease. However, there has not yet been a systematic analysis of the prevalence of hyperuricemia in China.

**Methods:**

Epidemiological investigations on hyperuricemia in China published in journals were identified manually and on-line by using CBMDISC, Chongqing VIP database and CNKI database. Those Reported in English journals were identified using MEDLINE database. Selected studies had to describe an original study defined by strict screening and diagnostic criteria. The fixed effects model or random effects model was employed according to statistical test for homogeneity.

**Results:**

Fifty-nine studies were selected, the statistical information of which was collected for systematic analysis. The results showed that the pooled prevalence of hyperuricemia in male was 21.6% (95%CI: 18.9%-24.6%), but it was only 8.6% (95%CI: 8.2%-10.2%) in female. It was found that thirty years was the risk point age in male and it was fifty years in female.

**Conclusions:**

The prevalence of hyperuricemia is different as the period of age and it increases after 30 years in male and 50 in female. Interventions are necessary to change the risk factors before the key age which is 30 years in male and 50 in female.

## Background

Hyperuricemia (HU) is a result of multifactor interactions including gender, age, genetic and environmental factors. Classically, the following conditions are associated with HU: alcoholism, obesity, hypertension, dyslipidemia, hyperglycemia, diabetes mellitus, lithiasis, renal failure, and medication use (diuretics, cyclosporine, low-dose aspirin) [1]. In the past several decades, the prevalence varied greatly and appeared to be increasing. There was lots information that demonstrated the importance of serum uric acid to the clinical prognosis, so the importance of HU is increasing. It reported that 18.8% of the patients with HU developed into gout in a 5 year follow-up [2]. Independent association between HU and cardiovascular disease has been found in many studies [3,4]. Hyperuricemia has been reported to be associated with several components of metabolic syndrome (MetS) and authors have postulated that increased concentrations of uric acid may be another important component of the syndrome [5].

With rapid economic development, possibility of improved nutrition and promotion of successful heath and medical care programs in China, life expectancy has been prolonged and the elderly population has increased; thus prevention and control of chronic diseases have become more important than before. Hyperuricemia may induce many complications, such as chronic gout, distortion of joint and renal failure, which may increase medical care costs. Therefore, it is important to study the hyperuricemia in China, in all developing countries, even in the whole world.

## Methods

### Search strategy

Studies were identified from the following electronic databases: CBMDISK, Chongqing VIP, CNKI and MEDLINE, using the terms 'hyperuricemia', 'HU' and 'prevalence'. No attempt was made to retrieve unpublished studies. The study did not include epidemiological studies in the areas of Hong Kong, Macao and Taiwan, because they are different from the Chinese mainland in the cultural activity and socioeconomic status and hence the prevalence of hyperuricemia and gout in those areas would be different from the Chinese mainland.

### Inclusion and exclusion criteria

In order to meet the analysis requirements and reduce deviation, selected studies fulfilled the following criteria: (i) case collection based on field survey; (ii) the study based on population samples rather than volunteers; (iii) There should be validated diagnostic criteria and accurate study dates; (iv) If there were many articles based on the same sample, only the one that reported the most detailed data was included. It was confirmed that all articles had the same diagnostic criteria. Studies were excluded if we could not obtain information necessary for the computation of prevalence in different sex and age from the articles or the authors.

### Quality of the studies

We accessed the quality of studies using the framework suggested by the Cochrane Collaboration. For the inclusion decision, quality assessment was carried out independently by three reviewers. If two of them or three agreed, the study can be included to the meta-analysis. The data from all included studies were clearly tabulated, and deviations were taken into account and identified during the quality assessment stage.

### Data analysis

We used a published systematic analysis technique to calculate the pooled prevalence of hyperuricemia and gout from all eligible studies. Summary of prevalence estimates were obtained using fixed-effects or random-effects meta-analysis which determined by I^2^. Statistical heterogeneity was assessed through I^2 ^statistic and its values of 25%, 50% and 75% correspond to low, moderate and high heterogeneity. The date which was low heterogeneity was chose the fixed-effects meta-analysis and others were chose random-effects meta-analysis. Subgroup analysis including sexes, ages and areas was also performed.

## Results

**Figure 1:**
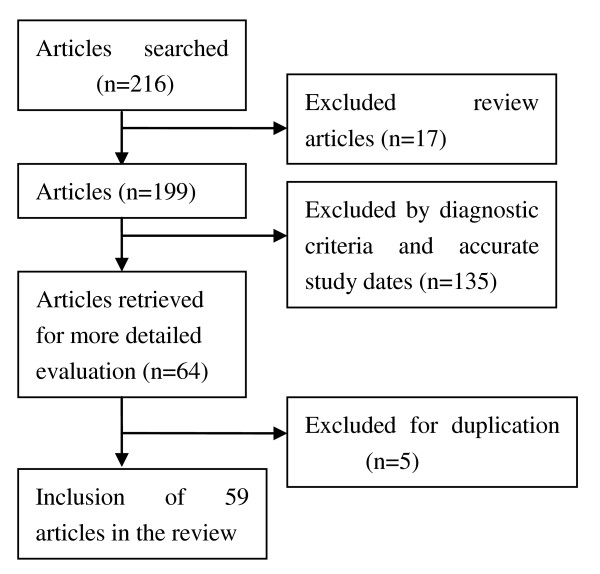
**Flow of information through the different phases of a systematic review**.

**Figure 2:**
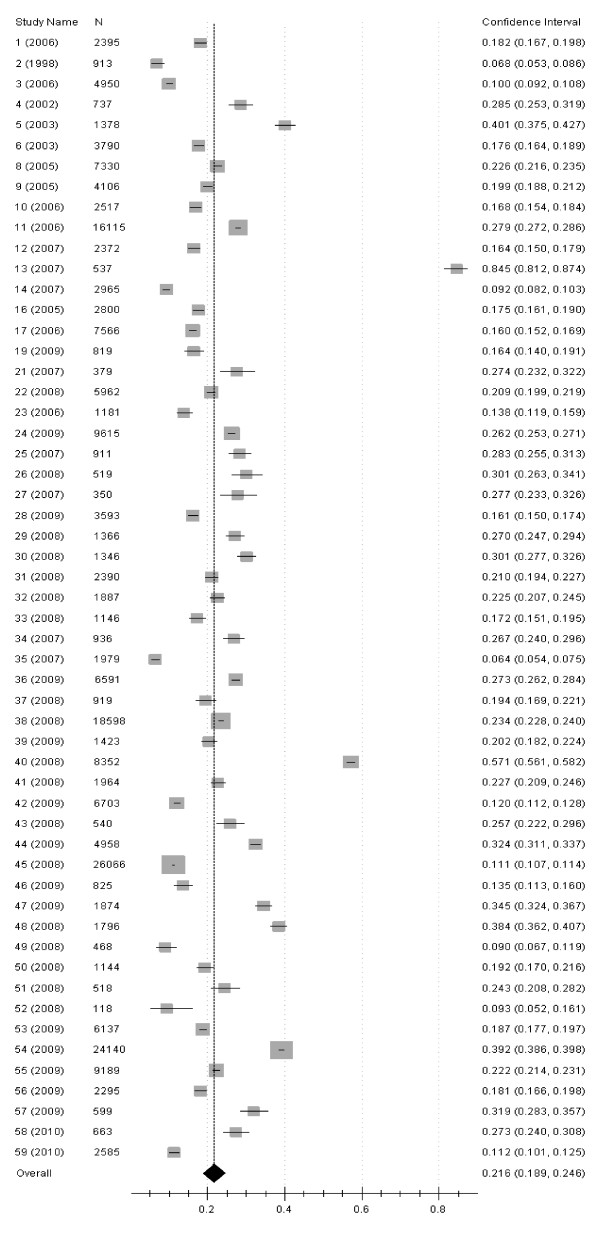
**Forest plot of the studies for the male**.

**Figure 3:**
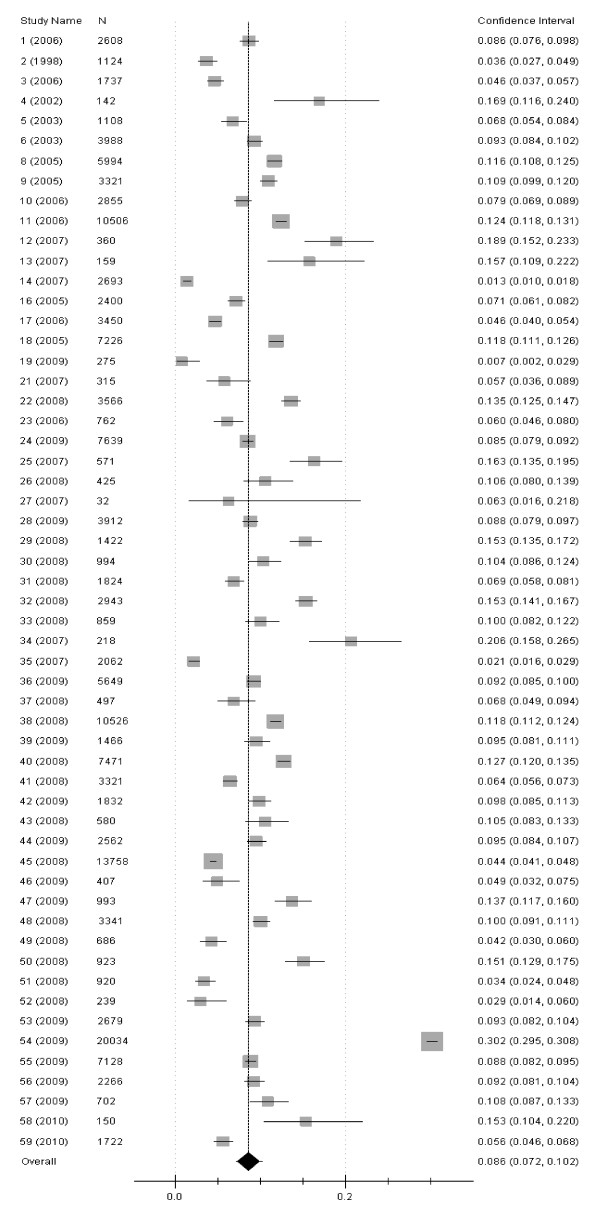
**Forest plot of the studies for the female**.

Figure 1 summarized the process of identifying eligible epidemiological studies. There were 59 [6-64] studies left after the quality assessment. Table 1 showed the characteristics of the studies, which covered 23 provinces in the review. The prevalence of HU and 95%CI in male and female were calculated separately for each study, also the sample size and published years can be found (Figure 2, Figure 3). The male population of 223,315 was investigated, and cases of 52,998 HU were selected. It was 165,620 in female, and cases of 19,586 HU were selected. The pooled prevalence of hyperuricemia in male was 21.6% (95%CI: 18.9%-24.6%) and it was only 8.6% (95%CI: 8.2%-10.2%) in female (Table 2), it was also found that the prevalence in female was lower than that in male in every age group. Table 2 also showed the prevalence of hyperuricemia in different gender and area. **Heterogeneity of the analysis was **moderate. The prevalence ranged from 8.4% to 8.6% in female, and it ranged from 19.6% to 26.8% in male. Table 3 demonstrated the prevalence of hyperuricemia in different age and area. It was found that thirty was the risk point age in male and it was fifty in female. The prevalence of female in northern and eastern China was 2.6% in ~30 age group, and it was high to 31.2% in western China of male in 51-60 age group.

**Table 1:** Characteristics of the studies

		Study design						
			
**NO**.	First author & year published	Age	Location (Western/Eastern) (Northern/Southern)	Survey date	Diagnostic criterion (μmol/L) (Male/Female)	Hyperuricemia no.(Male/Femal)	**Subjects no**. (Male/Female)	Prevalence (%)(Male/ Female)
1[6]	Miao et al.(2006)	20-80	Shandong(E)	May 1995 - Aug 1996	>420/>350	435/225	2395/2608	18.16/8.63

2[7]	Du et al.(1998)	≥15	Shanghai(E)	Nov 1996 - Aug 1997	>417/>357	62/41	913/1124	6.79/3.65

3[8]	Huang et al.(2006)	≥20	Jiangsu(E)	Jan 2000-Apr 2006	≥416.5/≥357	493/80	4950/1737	9.96/4.61

4[9]	Li et al.(2002)	36-90	Henan(E)	May 2000	>416/>357	210/24	737/142	28.49/16.90

5[10]	He et al.(2003)	20-72	Sichuan(W)	2002	≥420/≥360	552/75	1378/1108	40.06/6.77

6[11]	Shao et al.(2003)	≥20	Jiangsu(E)	Dec 2002-Mar 2003	≥417/≥357	668/370	3790/3988	17.63/9.28

7[12]	Wang et al.(2004)	≥40	Liaoning(E)	-----	>416/>357	192	1000	19.2

8[13]	Yu et al.(2005)	21-83	Guangdong(E)	Jan 2003-Mar2004	≥417/≥357	1655/697	7330/5994	22.59/11.63

9[14]	Yu et al.(2005)	22-81	Guangdong(E)	Jan 2003-June2004	≥417/≥357	819/363	4106/3321	19.95/10.93

10[15]	Zhang et al.(2006)	20-91	Shandong(E)	Mar 2003-Dec 2004	>416.36/>356.88	424/225	2517/2855	16.85/7.88

11[16]	Gu et al.(2006)	20-80	Guangdong(E)	2004	≥417/≥357	4496/1304	16115/10506	27.90/12.41

12[17]	Zhang et al.(2007)	28-88	Gansu(W)	2004	>440/>350	389/68	2372/360	16.40/18.89

13[18]	Luan et al.(2007)	21-72	Tibet(W)	Oct 2004 - Dec 2004	≥440/≥360	454/25	537/159	84.54/15.72

14[19]	Yao et al.(2007)	≥18	Shanghai(E)	Oct 2004 - June 2005	≥417/≥357	273/36	2965/2693	9.21/1.34

15[20]	Li et al.(2007)	20-59	Ningxia(W)	Jan 2004 - Dec 2005	>420/>350	410	9358	4.38

16[21]	Zeng et al.(2005)	18-85	Guangxi(W)	Jan 2004 - Aug 2005	≥417/≥357	490/170	2800/2400	17.50/7.08

17[22]	Mao et al.(2006)	≥20	Zhejiang(E)	Apr 2004-Dec2004	≥416/≥357	1214/160	7566/3450	16.05/4.64

18[23]	Diao et al.(2005)	20-79	Guangdong(E)	June 2004-June 2006	>357	0/853	0/7226	0/11.80

19[24]	Li et al.(2009)	22-60	Qinghai(W)	2004-2007	>420/>350	134/2	819/275	16.36/0.73

20[25]	Wu et al.(2007)	21-67	Zhejiang(E)	2005-2006	≥416/≥357	250	1492	16.76

21[26]	Sun et al.(2007)	20-70	Xinjiang(W)	Jan2005 - Dec2005	>417/>357	104/18	379/315	27.44/5.71

22[27]	Xie et al.(2008)	18-92	Chongqing(W)	June2005 - Dec2005	>380/>300	1244/483	5962/3566	20.87/13.54

23[28]	Fang et al.(2006)	20-90	Beijing(E)	Sept2005 - Dec2005	≥416.4/≥356.9	163/46	1181/762	13.80/6.04

24[29]	Cao et al.(2009)	>20	Zhejiang(E)	2005-2007	≥417/≥357	2516/651	9615/7639	26.17/8.52

25[30]	Wu et al.(2007)	19-87	Guangdong(E)	------	≥417/≥357	258/93	911/571	28.32/16.29

26[31]	Li et al.(2008)	>60	Guangdong(E)	2006	>420	156/45	519/425	30.06/10.59

27[32]	Jin et al.(2007)	26-57	Jilin(E)	------	≥408/≥357	97/2	350/32	27.71/6.25

28[33]	Chen et al.(2009)	>20	Yunnan(W)	Jan 2006 - Dec 2006	>420/>350	580/343	3593/3912	16.14/8.77

29[34]	Wu et al.(2008)	>16	Guangdong(E)	Nov2006-Feb2007	≥417/≥357	369/217	1366/1422	27.01/15.26

30[35]	Zeng et al.(2008)	22-79	Hunan(W)	Dec 2006 - Jan 2007	≥417/≥357	405/103	1346/994	30.09/10.36

31[36]	Wanget al.(2008)	20-78	Heilongjiang(E)	Feb2006 - Jan 2008	≥417/≥357	502/125	2390/1824	21.00/6.85

32[37]	Tian et al.(2008)	≥35	Shanghai(E)	Mar2006 - Sept2006	>420/>350	425/451	1887/2943	22.52/15.32

33[38]	Wei et al.(2008)	20-70	Hebei(E)	May2006 - Dec 2006	≥420/≥350	197/86	1146/859	17.19/10.01

34[39]	Deng et al.(2007)	41-93	Liaoning(E)	Sept2006 - Nov2006	>416/>339	250/45	936/218	26.71/20.64

35[40]	Wen et al.(2007)	35-64	Shandong(E)	Sept2006 - Dec 2006	≥417/≥357	126/44	1979/2062	6.37/2.13

36[41]	Zeng et al.(2009)	≥20	Zhejiang(E)	2006-2007	≥417/≥357	1797/520	6591/5649	27.26/9.21

37[42]	Zhong et al.(2008)	22-92	Hunan(W)	2007	≥420/≥360	178/34	919/497	19.37/6.84

38[43]	Zheng et al.(2008)	20-92	Guangdong(E)	2007	≥420/≥350	4355/1239	18589/10526	23.43/11.77

39[44]	Huang et al.(2009)	60-90	Shanghai(E)	2007	≥420/≥360	288/139	1423/1466	20.24/9.48

40[45]	Chen et al.(2008)	20-90	Chongqing(W)	2007	≥417/≥357	4772/951	8352/7471	57.14/12.73

41[46]	Liu et al.(2008)	≥60	Zhejiang(E)	Oct2007 - Feb2008	>420	445/213	1964/3321	22.66/6.41

42[47]	Jia et al.(2009)	20-101	Hebei(E)	Jan2007-Nov2007	>401.52/>249.20	802/180	6703/1832	11.96/9.83

43[48]	Han et al.(2008)	19-87	Beijing(E)	Jan2007-Nov2007	≥417/≥357	139/61	540/580	25.74/10.52

44[49]	Yuan et al.(2009)	20-85	Zhejiang(E)	Nov 2007 - Dec2007	≥417/≥357	1607/243	4958/2562	32.41/9.48

45[50]	Gao et al.(2008)	18-94	Anhui(W)	Jan2007 - Dec 2007	>429/>340	2883/609	26066/13758	11.06/4.43

46[51]	Wu et al.(2009)	23-59	Guangxi(W)	Jan2007 - May2009	>420/>360	111/20	825/407	13.45/4.91

47[52]	Yang et al.(2009)	24-67	Tibet (W)	Jan2007 - May2009	≥417/≥357	646/136	1874/993	34.53/13.70

48[53]	Wang et al.(2008)	45-96	Zhejiang(E)	Jan2007 - June 2007	≥420/≥360	690/335	1796/3341	38.42/10.03

49[54]	Quan et al.(2008)	32-77	Jilin(E)	Mar 2007	≥420/≥350	42/29	468/686	8.97/4.23

50[55]	Liu et al.(2008)	27-82	Liaoning(E)	Apr 2007 - Apr 2008	>420/>350	220/139	1144/923	19.23/15.06

51[56]	Wang et al.(2008)	>16	Zhejiang(E)	May 2007	>420/>360	126/31	518/920	24.32/3.37

52[57]	Ding et al.(2008)	21-61	Jiangsu(E)	May 2007	>420/>340	11/7	118/239	9.32/2.93

53[58]	Zhang et al.(2009)	20-90	Guangdong(E)	Sept2007-Aug2008	≥417/≥357	1147/248	6137/2679	18.69/9.26

54[59]	Huang et al.(2009)	20-80	Fujian (E)	Jan2008 - Sept2008	>416/>339	9458/6046	24140/20034	39.18/30.18

55[60]	Li et al.(2009)	30-69	Guangdong(E)	Mar2008 - Oct2008	≥428/≥340	2043/629	9189/7128	22.23/8.82

56[61]	Wang et al.(2009)	60-98	Beijing(E)	July2008 - June2009	≥417	416/208	2295/2266	18.13/9.18

57[62]	Wang et al.(2009)	19-65	Zhejiang(E)	Aug2008	≥417/≥357	191/76	599/702	31.89/10.83

58[63]	Cao et al.(2010)	>20	Hainan(E)	Sept 2008-Nov 2008	≥417/≥350	181/23	663/150	27.30/15.33

59[64]	Jiang et al.(2010)	20-93	Beijing(E)	Mar2009 - Sept2009	≥417/≥357	290/96	2585/1722	11.22/5.57

**Table 2:** The prevalence of hyperuricemia in different gender and area

China	Gender	Case/Total (No. of Studies)	Pooled Estimate (%)	95%CI(%)	Heterogeneity
					I2 P-value
Northern China	Male	8923/59413(n = 21)	19.6	15.7-24.1	49.8% 0.00
	
	Female	2393/35231(n = 21)	8.4	6.6-10.5	49.2% 0.00

Southern China	Male	44075/163902(n = 34)	22.7	19.6-26.1	49.9% 0.00
	
	Female	17289/132111(n = 35)	8.8	7.1-10.8	49.9% 0.00

Eastern China	Male	40056/166093(n = 41)	20.1	17.8-22.6	49.8% 0.00
	
	Female	16645/131127(n = 42)	8.6	7.0-10.6	49.8% 0.00

Western China	Male	12942/57222(n = 14)	26.8	17.3-39.0	50.0% 0.00
	
	Female	3037/36215(n = 14)	8.5	6.5-11.2	49.5% 0.00

Total	Male	52998/223315 (n = 55)	21.6	18.9-24.6	49.9% 0.00
	
	Female	19586/165620 (n = 55)	8.6	7.2-10.2	49.8% 0.00

**Table 3:** The prevalence of hyperuricemia in different age

China	Gender	Age					
		
		~30	31-40	41-50	51-60	61-70	>70
Northern China	Male	11.6(8.8-15.1)	16.4(12.7-20.9)	20.9(16.0-26.7)	21.0(14.2-29.9)	17.3(14.4-20.6)	17.9(13.8-23.0)
	
	Female	2.6(1.5-4.5)	3.8(2.4-5.9)	7.6(5.5-10.2)	14.3(9.1-21.7)	13.2(9.5-18.2)	20.2(14.4-27.6)

Southern China	Male	16.8(11.4-24.2)	24.4(16.3-34.9)	24.9(19.6-31.2)	23.5(19.5-27.9)	22.6(19.7-25.7)	27.2(22.5-32.4)
	
	Female	3.1(1.8-5.4)	3.6(1.7-7.6)	6.2(3.5-10.7)	15.1(11.1-20.2)	19.8(13.6-27.8)	25.3(17.6-35.0)

Eastern China	Male	13.1(9.5-17.7)	17.4(13.4-22.2)	20.4(16.7-24.7)	19.6(16.4-23.2)	19.9(17.3-22.8)	24.2(21.0-27.7)
	
	Female	2.6(1.5-4.6)	3.5(2.6-6.7)	6.8(4.2-10.6)	12.4(9.4-16.2)	15.3(10.7-21.6)	22.4(16.2-30.0)

Western China	Male	17.5(6.3-40.1)	29.9(8.3-67.0)	31.0(18.8-46.4)	31.2(18.9-46.9)	25.6(18.7-27.0)	24.6(10.3-48.1)
	
	Female	4.7(3.4-6.5)	4.2(2.6-6.7)	7.5(5.2-10.6)	22.9(11.7-39.8)	24.6(16.1-35.6)	31.1(24.5-38.6)

Total	Male	14.2(10.4-19.2)	20.1(15.3-26.0)	22.9(18.9-27.5)	22.3(18.8-26.2)	23.3(18.0-24.9)	24.1(20.6-28.0)
	
	Female	2.8(1.8-4.5)	3.5(2.0-6.2)	6.6(4.5-9.7)	14.7(11.5-18.6)	16.8(12.5-22.4)	23.4(17.7-30.4)

Note: The I^2 ^of all studies in the table ranged from 45% to 50%.

## Discussion

The prevalence of hyperuricemia varies in different populations and areas. In Turkey [65], one study reported that 19% of the men and 5.8% of the women had hyperuricemia and the overall prevalence of hyperuricemia was 12.1% in the urban population. In Nepal [66], 3794 people which were from Chitwan districts were investigated, and the prevalence of hyperuricemia was 21.42%. In Seychelle [67], the cross-sectional health examination survey based on a population random sample which included 1011 subjects aged 25 to 64 years showed that the prevalence of hyperuricemia was 35.2% and 8.7% in men and women, respectively. In Thailand [68], an across-sectional study of 1381 patients who firstly participated in annual health examinations during the period of July 1999 through February 2000 reported that the prevalence of hyperuricemia was 10.6%, but it was 18.4% and 7.8% in men and women, respectively. In Java [69], the prevalence of hyperuricemia was investigated by a survey of a total population of 4683 rural adults and the result was 24.3%. In United States [70], the prevalence rate of asymptomatic hyperuricemia in the general population was estimated at 2-13%. The prevalence of gout and/or hyperuricemia increased about 2 cases per 1000 enrollees over 10 year (1990-1999) in the overall population. In Japan [71], a total of 9,914 individuals (6,163 men and 3,751 women aged from 18 to 89 years) who were screened at Okinawa General Health Maintenance Association was screened. The result showed that the prevalence of hyperuricemia was 25.8% and it was 34.5%, 11.6% in men and women respectively. In New Zealand [72], hyperuricemia was more common in Maori men (27.1%) than in European men (9.4%) and in Maori women (26.6%) than in European women (10.5%). In Saudi Arabia [73], the prevalence of hyperuricemia was only 8.84%. In Taiwan island of China [74], the prevalence of hyperuricemia was high to 49.4% in Ayatals, but it was only 27.4% in non-aborigines.

From the analysis, it was found that age and sex affected the serum uric acid levels and the prevalence of hyperuricemia:

### The factor of age

It was found that the prevalence of hyperuricemia increased with the age in male and female. The prevalence was higher in male who were after 30 years old than that younger. But the point age was 50 in female. The physiologic and economic reasons may explain this difference. After 30 years old, the male would have a stable family and career. In female, the influence of sexual hormones may explain the point age. Young children of both sexes have equally low urate levels, so the prevalence is low. The study of Katrine demonstrated that the 45-64 age group was higher prevalence compared with the 18-44 age group [75]. Vitool's study showed that the prevalence were 4.3% and 1.3% in men and women, who were younger than 18 years, but it increased to 17.4% and 15.4% in the men and women from 30 to 39 [68]. A study about elderly people in Taiwan reported that Men at age 65 to 69 had the highest proportion of hyperuricemia which was 69.8%, but woman at age more than 80 had the higher prevalence which was 50% [76].

### The factor of sex

From the previous studies, it was found that serum uric acid levels were higher in men than in women, but it tended to be consistent between man and woman after the age of 50 [77,78]. The study of Gordon explained it that serum uric acid level increased after the menopause in females which attributed to the influence of sexual hormones [79]. The results of the study showed that male subjects had a higher prevalence of hyperuricemia than women, which was in line with findings of many studies from different countries [65-74].

### Health Education and life customs

From the result of meta-analysis, it was found that the prevalence in different age of southern China was higher than that in northern China and the prevalence in western was higher than that in eastern, especially in male. The reason for that may be different life customs. In southern China, the mainly food is rice and it is sweat in northern China. In the eastern China, the health service is better than that in western China. More health educations were carried out and the people had more health knowledge in eastern China, which may affect the prevalence of hyperuricemia. The reasons for the difference in prevalence need further research.

## Conclusions

In conclusion, aging trend is more and more serious in China, even all the word, and the prevalence of hyperuricemia is higher in elderly. It was found that urate levels correlate with many recognized cardiovascular risk factors, including hypertension, diabetes mellitus, hypertriglyceridemia, obesity and insulin resistance. Multiple Risk Factor Intervention Trial (MRFIT) database showed that hyperuricemia was an independent risk factor for acute myocardial infarction [75]. The Italian Progetto Ipertensione Umbria Monitoraggio Ambulatoriale (PIUMA) study showed that serum urate levels in the highest quartile were associated with increased risk of all cardiovascular events (relative risk [RR] = 1.73) and fatal cardiovascular events (RR = 1.96) compared with urate levels in the second quartile[76]. So it is important to control the prevalence in elderly. Interventions are necessary to change the risk factors before the key age which is 30 years in male and 50 in female. At the same time, intervention to high risk group is urgent.

In China, most of the studies concerned the eastern, especially in the urban areas, but it is necessary to study the western of China and rural areas. The cohort study with larger sample is necessary. This article only provides the narrowing window of hyperuricemia in China.

## Competing interests

The authors declare that they have no competing interests.

## Authors' contributions

LB and WT gave the biggest contributions to the passage; JRY gave the point about the passage; ZHHN, YWW, YHP, LCX, YJ helped to analysis and interpret data; NHW helped to revise the language problems. All authors read and approved the final manuscript.

## Pre-publication history

The pre-publication history for this paper can be accessed here:

http://www.biomedcentral.com/1471-2458/11/832/prepub
